# Winning the battle of intestinal peace with *Bacillus*—a multifaceted approach to animal health, immunity, and future applications in monogastric livestock production

**DOI:** 10.3389/fmicb.2025.1711747

**Published:** 2025-12-15

**Authors:** Nuria Vieco-Saiz, Olga Lemâle, Nicholas P. Evans, Wanderley M. Quinteiro-Filho, Amine Mellouk, Jessika Consuegra, Haitham Yakout, Tim Goossens

**Affiliations:** 1 European Laboratory of Innovation Science & Expertise (ELISE), Adisseo France S.A.S., Saint Fons, France; 2 Adisseo NL B.V., Raamsdonksveer, Netherlands; 3 Adisseo USA Inc., Alpharetta, GA, United States; 4 Adisseo Brasil Nutriçao Animal Ltda, São Paulo, Brazil; 5 Adisseo, Sint-Niklaas, Belgium

**Keywords:** probiotic, *Bacillus*, livestock, monogastric, health

## Abstract

This review focuses on the application of *Bacillus*-based probiotics in livestock production, emphasizing their potential to enhance animal health, growth performance, and welfare through modulation of the gut microbiota, immune function, and nutrient absorption. *Bacillus* probiotics, particularly spore-forming strains, offer advantages such as stability during feed processing or the ability to survive gastrointestinal conditions, germinate and produce beneficial metabolites in the intestine. The mechanisms by which *Bacillus* probiotics exert their effects include influencing microbial communities, producing bioactive compounds, and strengthening gut barrier integrity, which together lead to improved digestive health and resistance to pathogens. Additionally, the challenges in standardizing their effects and identifying reliable biomarkers for evaluating probiotic efficacy or the axes with other organs are highlighted. Overall, *Bacillus* probiotics are seen as promising, sustainable alternatives to antibiotics with significant potential for future research to optimize their use and understand their mechanisms within the context of animal production and health.

## 1 Introduction

Probiotics are defined by the Food and Agriculture Organization of the United Nations and the World Health Organization (FAO/WHO) as “live microorganisms that, when administered in adequate amounts, confer a health benefit on the host.” This definition allows for a broad range of microbes to be used in animal feed, as a means to improve animal health and performance (Hill et al., 2014).

The use of health-supporting feed additives such as probiotics has gained increasing interest in the poultry and swine industry and is driven by the challenge in livestock production to stimulate efficiency in raising high-performing animals while becoming less dependent on antimicrobials. Apart from preventing and treating diseases, veterinary drugs have been applied as antibiotic growth promoters. However, the increasing number of antibiotic-resistant bacteria, which are a danger to both animal and human health, has accelerated the quest for alternatives to antibiotics that are able to stimulate growth and to make the animal more resilient to production challenges (Popov et al., 2021; Luise et al., 2022; Zhang et al., 2023). Probiotics came into the picture as a non-antimicrobial candidate to improve animal productivity, because evidence from human research had suggested that their mechanisms to improve gut health were conserved in production animals, and could be produced and applied in animal feed in a cost-effective way (Zommiti et al., 2020).

Although the most common probiotics are non-spore-forming strains like *Lactobacillus* and *Bifidobacterium*, spore-forming bacteria, like *Bacillus subtilis* and *B. amyloliquefaciens*, are now being utilized more frequently, due to several key advantages (Popov et al., 2021). Hence, in this review, we will first give an overview of commonly used probiotic bacteria in poultry and swine production. Secondly, we will focus on *Bacillus*-based probiotics, summarizing our current understanding of their underlying modes of action in pigs and poultry and describing the evidence for their potential role in contributing to the specific Sustainable Development Goals (SDGs) as established by the United Nations. Thirdly, research gaps and potential future research directions will be discussed.

## 2 Probiotics used in poultry and swine industry

### 2.1 Probiotics in poultry and swine production

Various dietary strategies have been applied as natural growth promoters in the poultry and swine industry, such as prebiotics, probiotics, enzymes, antimicrobial peptides (AMP), organic acids, bacteriophages, synbiotics, metal, clay, hyperimmune egg yolk IgY, phytogenics and most recently, postbiotics (Humam et al., 2019; Halder et al., 2024). Probiotics are, by definition, the opposite of antibiotics, as they rely on the growth of bacteria to exert a beneficial and revitalizing function in the digestive tract (Lilly and Stillwell, 1965). When adequately consumed, these living microorganisms can modulate gut health, performance, and animals’ overall health. Benefits of dietary probiotic consumption therefore exceed the typical nutritional edge to go beyond to complicated interactions with gut microbiota, many metabolic processes, and immune responses, as explained below (Halder et al., 2024; Qadri et al., 2024).

### 2.2 Probiotic genera

**Figure 1:**
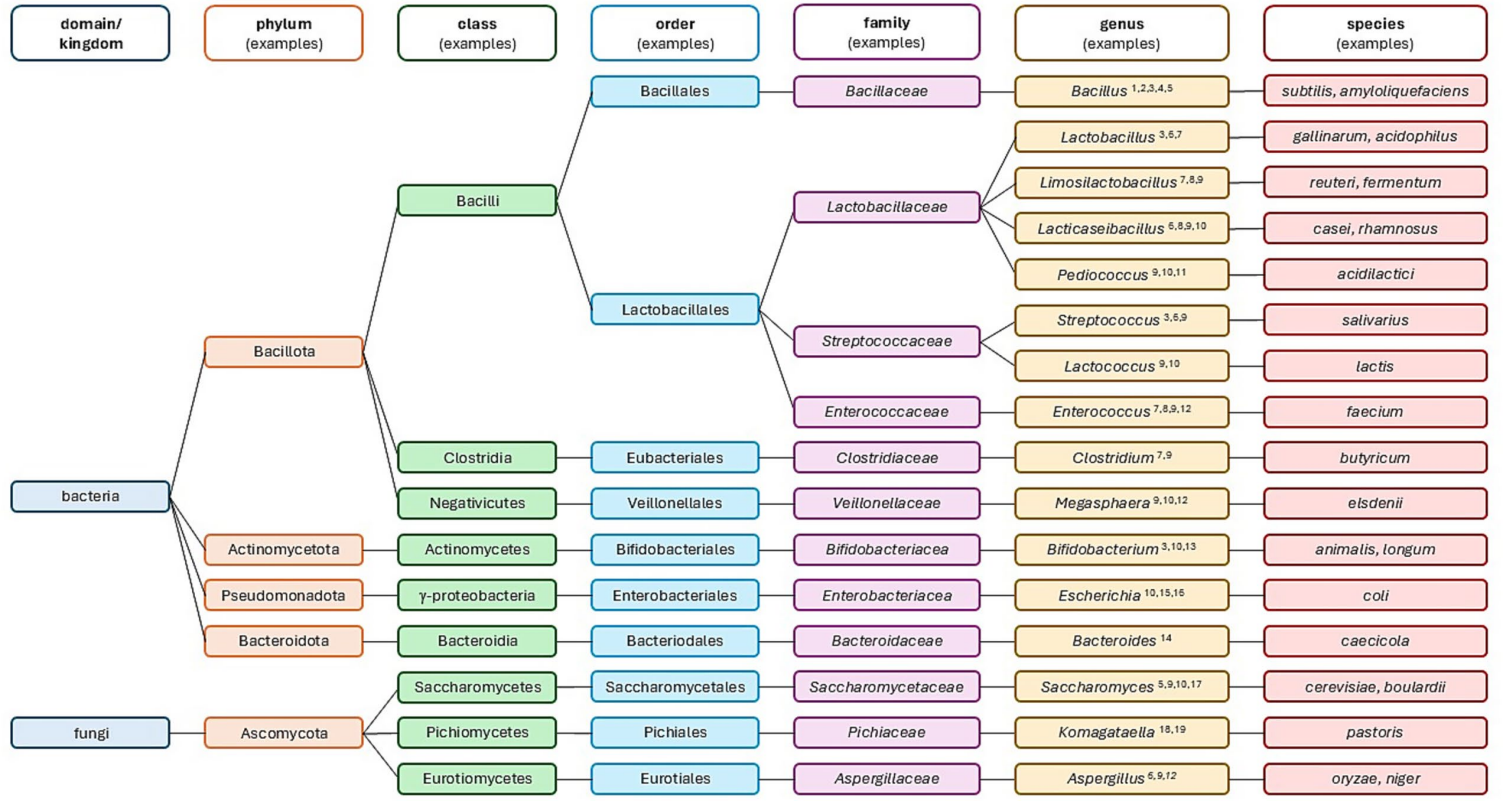
Taxonomic classification of examples of bacterial genera used as probiotic in poultry and swine production. ^1^Stefanello et al. (2025); ^2^Jacquier et al. (2019); ^3^Rahman et al. (2014); ^4^Wongsamart et al. (2025); ^5^Barbosa et al. (2024); ^6^Daşkiran et al. (2012); ^7^Halder et al. (2024); ^8^Yang et al. (2015); ^9^Liao et al. (2015); ^10^Anee et al. (2021); ^11^Wideman et al. (2012); ^12^Seo et al. (2010); ^13^Pedroso et al. (2013); ^14^Papouskova et al. (2023); ^15^Wu et al. (2023); ^16^Mourand et al. (2021); ^17^Elghandour et al. (2020); ^18^Peng et al. (2014); ^19^de Los Santos et al. (2018).

Selecting a microbial strain is the most critical task when developing a probiotic product. The taxonomic classification may already encompass information about physiology, ecological niche preference, and metabolic characteristics of that particular strain. These specific characteristics, together with the intended application of the probiotic product, will crucially dictate the choice of microbial strain (Morelli, 2007). The most widely used probiotic genera in animal production consists of strains from bacterial and fungi genera (Figure 1).

Regardless of how the probiotic product will be applied, all candidate strains must be generally recognized as safe (GRAS), non-toxic, non-pathogenic, beneficial to the host and able to withstand gastrointestinal acid and bile salts (Fuller, 1989; Ezema, 2013).

### 2.3 Selecting probiotic strains

The targeted application method of a probiotic product has a critical impact on the choice of probiotic strains. A probiotic can be supplemented to the diet of poultry and swine throughout their entire production cycle. In that case, there is no need to select gut colonizing bacteria, which allows selection of strains that are not natural inhabitants of the chicken or swine gut, but have metabolic characteristics different from those of the endogenous microbiota (Le Duc et al., 2004).

**Figure 2:**
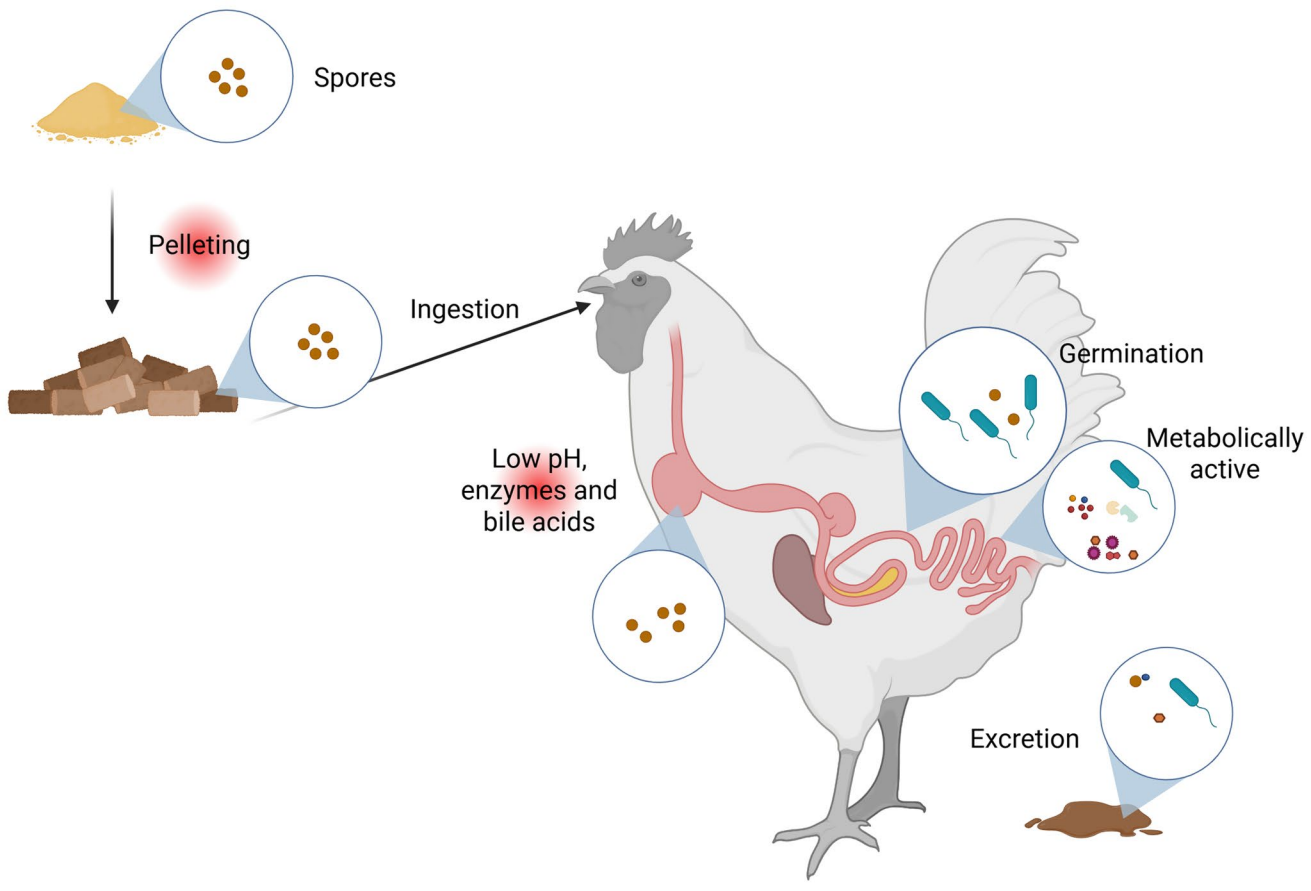
Fate of probiotic *Bacillus* spores and vegetative cells in the gastrointestinal tract. Spores are added to the feed and have to survive pelleting and proximal gastrointestinal tract conditions (such as low pH, digestive enzymes, bile acids) before they can germinate in the intestine. After germination, *Bacillus* can produce and secrete metabolites. Created in BioRender: https://BioRender.com/6b8zsaa.

The method of administration to the animal also has to be considered. Drinking water application is only possible if the probiotic product does not clog the nipples of the water line, and if bacterial overgrowth and biofilm formation in the drinking water lines are avoided. For liquid feed or mash feed, the product does not have to be heat-stable, but can have a fermentative effect (Khomayezi and Adewole, 2022). Probiotics that end up in pelleted feed, however, must be able to withstand higher temperatures and steam used in the pelleting process. Therefore, spore-forming bacteria are typically used for this purpose (Hong et al., 2005) (Figure 2).

Another factor to consider is the choice between a single probiotic strain or a mixture of several strains (Kwoji et al., 2021; Halder et al., 2024). Multi-strain products, which can be composed of different bacterial species, or a combination of bacteria and fungi (Timmerman et al., 2004) can have, at least in theory, a broader activity range than a single-cell product. However, demonstrating an additive or synergic effect of the selected strains is challenging, as it requires a series of dose–response trials *in vivo*, with various levels of different strain combinations. Even when making use of only a single species, thorough *in vitro* and *in vivo* analyses are warranted, as strains from the same species can have vastly different metabolic activities (Kruse et al., 2021; Kruse et al., 2022b).

Based on these selection criteria, certain bacterial genera have proven particularly well-suited for probiotic applications in animal nutrition. Among them, *Bacillus* species have received considerable attention and are discussed in more detail below.

### 2.4 *Bacillus* as probiotic

*Bacillus* species are one of those strains perfectly positioned as feed additives, mainly because of their stability as spore-forming bacteria (Schreier, 1993; Jiang et al., 2021). Sporulation is a process used by bacteria to protect themselves against environmentally dangerous and damaging factors, including heat, desiccation, and UV radiation (Setlow, 2006; Vilà et al., 2010). Hence, spores can tolerate different storage conditions, and they can withstand feed pelleting conditions and low gastric pH levels (Lorenzoni, 2010; Liao and Nyachoti, 2017) before they can germinate in the intestine to exert their beneficial functions (Figure 2).

The mode of action of *Bacillus* is based on different mechanisms, allowing a broad range of beneficial effects to the host. Once metabolically active in the intestine, *Bacillus* is able to produce several metabolites like enzymes, vitamins, and exopolysaccharides (Díaz-Cornejo et al., 2023). Furthermore, *Bacillus* can secrete antimicrobial compounds as well as compete with pathogens for essential resources (Caulier et al., 2019; Zhu et al., 2023).

Using probiotics based on *Bacillus* has shown beneficial effects on young pigs’ growth performance, nutrient absorption, intestinal structure, and immune response (Mun et al., 2021). There are many studies reporting reduction in inflammatory responses under heat stress, improved tight junctions’ integrity and influences on intestinal microbiota composition as a result of dietary supplementation of *Bacillus* species-based probiotics (Rhayat et al., 2019; Memon et al., 2022). Also, in lipopolysaccharide-challenged broilers, *B. pumilus* TS1 attenuated the inflammatory response and alleviated inflammatory injury. Furthermore, it enhanced growth performance, improved the intestinal microbial composition, and restored villus morphology, resulting in increased villus length (Liu et al., 2023).

While *Bacillus* species are known for their growth-promoting and health-supporting properties, it is essential to recognize that probiotic effects are strain-specific. As such, referring to a *Bacillus* as a probiotic requires careful assessment at the strain level to ensure its efficacy and relevance in a given context (Plaza-Diaz et al., 2014; Bernardeau et al., 2017).

In summary, the demonstrated benefits and robustness of *Bacillus* species provide a solid foundation for exploring in more detail the mechanisms through which these probiotics exert their positive effects on animal health and performance (Table 1).

**Table 1:** Overview of *Bacillus*-based probiotic activities.

Sections		*Bacillus* based probiotic	Evidences	References
Bioactive molecules production	Enzymes	*B. subtilis ED-3-7*	*In vitro*	Liu W. et al. (2025)
		*B. amyloliquefaciens* AM0938, JD17 *B. subtilis* AM1002	*In vitro*: 10^8^ spores/g feed	Latorre et al. (2014)
		*B. subtilis* ZJ12-1 *Ent. faecium* NCIMB 10415	*In vivo*: 10^8^ CFU/g feed	Shi et al. (2017)
		*B. velenzensis* YM1	*In vitro*	Feng et al. (2025)
	Antimicrobials	*B. subtilis* ATCC 6633	*In vitro*	Duitman et al. (1999)
		*B. subtilis* ATCC 21332	*In vitro*	Davis et al. (1999)
		*B. subtilis* LYS1	*In vivo*: 10^6^ CFU/g feed	Lee et al. (2023)
		*B. subtilis* ATCC 55422	*In vitro*	Butcher et al. (2007)
		*B. subtilis* PS-216	*In vitro*	Podnar et al. (2022) and Erega et al. (2021)
		*B. coagulans* *B. licheniformis* *B. pumilus* *B. subtilis*	*In vitro*	Buiatte et al. (2023)
		*B. subtilis* ATCC 6633	*In vitro*	Poormontaseri et al. (2017)
		*B. amyloliquefaciens* DSM7T, PTA-6507, NRRL B-50104, NRRL B-50013	*In vitro*	Medina Fernández et al. (2019)
	Related to host metabolism and immune modulation	*B. subtilus* sp. *suppress*	*In vitro*	Ghoneim et al. (2016)
		*B. subtilis* 3610, DS991	*In vitro*	Paynich et al. (2017)
		*B. subtilis* Z15	*In vitro*	Cao et al. (2023)
		*B. subtilis* DSM 29784	*In vivo*: 10^7^ CFU/g feed	Choi et al. (2021)
		*B. subtilis* ABP1	*In vitro*	Vicente-Gil et al. (2024)
		*B. subtilis* 168	*In vitro*	Domínguez Rubio et al. (2020)
Microbiota modulation		*B. subtilis* C-3102	*In vivo*: 10^6^ CFU/g feed	Jeong and Kim (2014)
		*B. subtilis* DSM 29784	*In vivo*: 10^5^ CFU/g feed	Jacquier et al. (2019)
		*B. subtilis* DSM 32540 *B. pumilus* DSM 32539	*In vivo*: 5 × 10^5^ CFU/g feed	He et al. (2020a)
		*B. subtilis* DSM 32324, DSM 32325 *B. amyloliquifaciens* DSM 25840	*In vivo*: 10^6^ CFU/g feed	Khan and Chousalkar (2021)
		*B. subtilis* *B. licheniformis* *Saccharomyces cerevisiae*	*In vivo*: 5 × 10^9^ CFU/g; 10^10^ CFU/g; 10^9^ CFU/g feed	He et al. (2019)
		*B. subtilis* *B. licheniformis*	*In vivo*: 10^6^ CFU/g feed	Kaewtapee et al. (2017)
		*B. subtilis* HGCC-1	*In vivo*: 10^8^ CFU/g feed	Li et al. (2025)
		*B. amyloliquefaciens* DSM 25840 *B. subtilis* DSM 25841	*In vivo*: 10^6^ CFU/g feed	Luise et al. (2019)
		*B. subtilis* CSL2	*In vivo*: 10^7^ CFU/g feed	Oh et al. (2017)
		*B. subtilis* DSM 32315	*In vivo*: 5 × 10^5^ CFU/g feed	Whelan et al. (2019)
Gut-associated immune system		*B. subtilis* DSM 32315 *B. velezensis* CECT 5940	*Ex vivo*	Larsberg et al. (2023)
		*B. subtilis* DSM 32315 *B. velezensis* CECT 5940	*Ex vivo*	Larsberg et al. (2024)
		*B. subtilis* DSM 29784	*In vitro*: 10^7^ CFU/mL	Rhayat et al. (2019)
		*B. subtilis* DSM 29784	*In vitro*: 10^7^ CFU/ml	Vieco-Saiz et al. (2024)
		*B. megaterium* SF185	*In vivo*: 10^9^ spores by gavage	Mazzoli et al. (2019)
		*B. subtilis* DSM 29784	*In vivo*: 10^5^ CFU/g feed	Keerqin et al. (2021)
		*B. subtilis* BG5, BYS2	*In vivo*: 10^6^ CFU/g feed	Guo et al. (2020)
		*B. subtilis* HB3	*In vivo*: 10^9^ CFU in 2 intramuscular injections	Lee et al. (2020)
		*B. subtilis* DSM 32540 *B. pumilus* DSM 32539	*In vivo*: 10^2^ CFU/g feed	He et al. (2020a)
		*B. subtilis*	*In vivo*: 10^6^ CFU/g feed	He et al. (2020b)
		*B. subtilis* HGCC-1	*In vivo*: 10^8^ CFU/g feed	Li et al. (2025)
		*B. amyloliquefaciens* DSM 25840 *B. subtilis* DSM 25841	*In vivo*: 10^6^ CFU/g feed	Luise et al. (2019)
		*B. subtilis* 14,823	*In vivo*: 10^6–7^ CFU/g feed	Khongthong et al. (2025)
		*B. subtilis* B. 747, B. 1999	*In vivo*: 0.05%	Wang et al. (2021)
		*B. subtilis*	*In vivo*: 10^6^ CFU/g feed	Wang et al. (2018)
Extraintestinal effects	Gut-liver axis	*B. licheniformis*	*In vivo*: 10^6^ CFU/g feed	Barba-Vidal et al. (2017)
		*Lactobacillus*; Yeast; *Bacillus*	Fermented feed	Gu et al. (2022)
	Gut-lung axis	*B. subtilis* 597	*In vivo*: 10^6^ CFU/g feed	Winther et al. (2024)
		6 *B. subtilis* strains	*In vivo*: 10^5^ CFU/g feed	Zuckermann et al. (2022)
		*B. subtilis* KC1	*In vivo*: 10^5^ CFU/g feed	Chen X. et al. (2023)
	Gut-brain axis	*B. velenzensis* ADS024	*In vitro*, *in vivo*: 5 × 10^8^ CFU by gavage	Acton et al. (2024)
		*B. subtilis* PXN21	*In vivo*: 10^8^ CFU by gavage	Rout et al. (2024)
		*B. licheniformis*	*In vivo*	Feng et al. (2023)
		*Bacillus clausii*; *Lactobacillus fermentum* NMCC-14	*In vivo*: 10^10^ CFU/mL/day	Rehman et al. (2022)
		*Bacillus coagulans Unique* IS-2	*In vivo*: 10^9^ CFU/day	Satti et al. (2023)
		*B. subtilis*	*In vivo*: 10^6^ CFU/g feed	Fu et al. (2023)
		*B. subtilis*	*In vivo*: 10^6^ CFU/g feed	Wang et al. (2018)

## 3 Modes of action of *Bacillus*

### 3.1 Bioactive molecules produced by *Bacillus*

**Figure 3:**
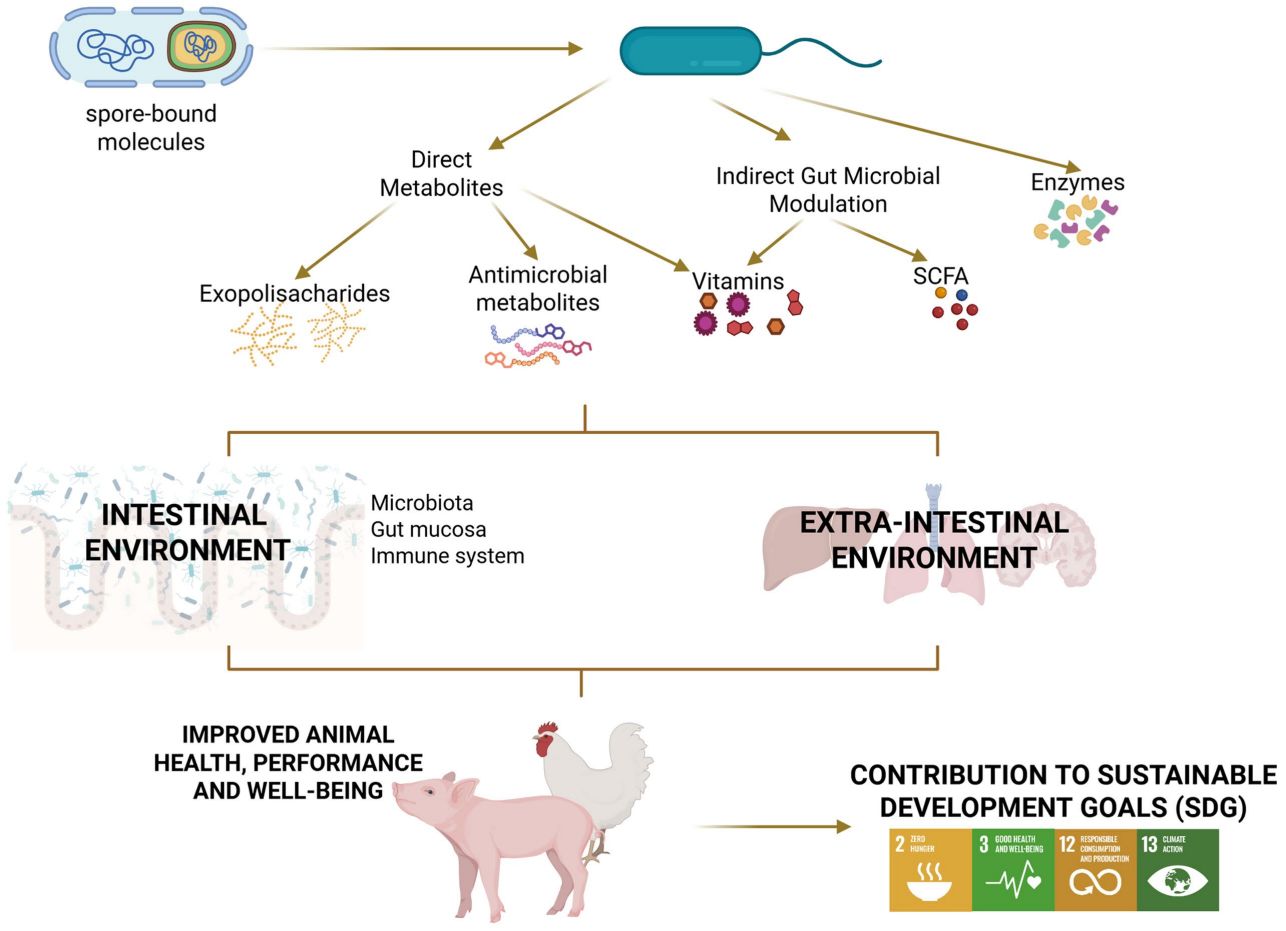
*Bacillus* probiotics secrete molecules that underly their beneficial effect. *Bacillus* probiotics can exert their effect through spore-bound or secreted molecules. These molecules can affect the intestinal environment and extra-intestinal organs either directly or indirectly through the modulation of the gut microbiota. The responses triggered by these molecules can improve animal health and contribute to sustainable development goals. Created in BioRender: https://BioRender.com/6b8zsaa.

*Bacillus* can instigate a wide range of probiotic actions, which is rooted in the multifariousness of its metabolic activities. *Bacillus* spp. secretes a vast range of bioactive molecules, such as enzymes, antimicrobial peptides, vitamins, and exopolysaccharides (Figure 3).

Being potent enzyme producers, *Bacillus* spp. secrete proteases, amylases, lipases, cellulases, and phytases (Latorre et al., 2015; Latorre et al., 2016). As feed materials for poultry and swine diets commonly include high levels of non-starch polysaccharides (NSP) and other antinutritional factors that are not efficiently digested by endogenous pancreatic enzymes (Knudsen, 2014), *Bacillus*-based products can be used to break down antinutritional compounds and enhance feed efficiency in animal nutrition (Latorre et al., 2015; Latorre et al., 2016). This can be achieved by applying *Bacillus* spp. directly to feed components, to pretreat raw materials like soybean meal, enhancing its nutritional profile and bioavailability (Shi et al., 2017). Fermentation of soybean meal has the additional advantage to be able to modulate the gut microbiota, leading to a decrease in cecal richness and diversity while promoting the growth of beneficial *Lactobacillus* populations (Liu W. et al., 2025). In addition, *Bacillus* spp. may modulate digestion in the intestinal tract by lowering digesta viscosity and restricting the amount of nutrients that are available for opportunistic pathogenic bacteria, such as *Clostridium perfringens* (Kogut and Arsenault, 2016). Other *Bacillus*-derived enzymes will increase gut health by their detoxifying activity, such as zearalenone lactonase, which is capable of degrading mycotoxins from feed materials (Feng et al., 2025; Song et al., 2025). *Bacillus* spp. also produce a diverse array of metabolites that contribute to their survival in their ecological niche, such as antimicrobial peptides like surfactin, bacillaene, macrolactin, bacilysin, and subtilosin A (Chen et al., 2015; Santos et al., 2018; Kruse et al., 2022b; Kruse et al., 2022a). These molecules can also have beneficial effects. Dietary supplementation of surfactin-producing *B. subtilis* LYS1, for example, has been shown to enhance growth performance and gut health in birds by promoting the development of intestinal villi (Lee et al., 2023). Similarly, bacillaene production by *B. subtilis* has been described to contribute to suppressing *Salmonella* growth (Patel et al., 1995; Podnar et al., 2022) and *Campylobacter jejuni* biofilm formation (Erega et al., 2021).

Beyond antimicrobial properties, *Bacillus-*derived compounds also play a crucial role in host metabolism and immune modulation. For instance, *Bacillus* exopolysaccharides enhance lipid metabolism, regulate blood glucose, and offer antioxidant benefits (Ghoneim et al., 2016). They also exhibit immunomodulatory effects by promoting anti-inflammatory M2 macrophages, which inhibit CD4^+^ and CD8^+^ T cells (Paynich et al., 2017).

Additionally, *Bacillus* spp. produce gut-derived nutrients and vitamins, like niacin, pantothenate, and hypoxanthine (Choi et al., 2021), which have gut-protective effects, by reducing pro-inflammatory pathways (AP-1, NF-κB), enhancing mucin production (MUC2 expression), stimulating cell proliferation, and strengthening epithelial barrier integrity against inflammation (Vieco-Saiz et al., 2024).

*Bacillus* produce extracellular vesicles (EVs) that are cell-derived membrane-surrounded vesicles carrying bioactive molecules that play a role in bacterial competition, survival, and host interactions (Bose et al., 2020). These EVs are proposed to facilitate communication between probiotics and the mammalian gastrointestinal tract (Domínguez Rubio et al., 2020). Their involvement in these processes highlights the immunomodulatory potential of Gram-positive bacterial EVs, particularly those derived from *Bacillus*, which have been shown to modulate both innate and adaptive immune functions (Vicente-Gil et al., 2024).

All these *Bacillus*-derived bioactive molecules can affect the gut microbiota, the intestinal epithelium, the gut-associated immune system, and extra-intestinal issues (described below), explaining the animal health and performance supporting effect of *Bacillus*-based probiotics. These bioactive molecules might contribute to animal health and performance through their modulation of the gut microbiota, the intestinal epithelium, the gut-associated immune system, and extra-intestinal tissues. The effect of *Bacillus*-based probiotics on these cells and tissues is described in more detail in the sections below.

### 3.2 Gut microbiota

*Bacillus* probiotics are widely studied for their effect on enhancing intestinal health through gut microbial modulation, by increasing beneficial bacteria, improving metabolite production, and reducing harmful microbial populations. More in detail, their effects on gut microbiota composition and diversity vary depending on study conditions, host species, and environmental factors.

In poultry raised under standard conditions, *Bacillus* supplementation has been linked to increased beneficial bacteria such as *Lachnospiraceae*, *Ruminococcaceae*, *Faecalibacterium* and *Lactobacillus* while reducing potentially harmful *Enterobacteriaceae* as demonstrated for *B. subtilis* CGMCC1921 and *B. subtilis* DSM 29784 (Guo et al., 2018; Choi et al., 2021). However, microbial diversity effects remain inconsistent, with some studies reporting increased richness, whereas others found no significant changes when using *B. subtilis* DSM 29784 or *B. subtilis* CSL2 (Oh et al., 2017; Jacquier et al., 2019). Under pathogenic conditions, *B. subtilis* CSL2 has also been shown to protect against infections like *Salmonella* Gallinarum by promoting beneficial microbial shifts and enhancing gut integrity (Oh et al., 2017). Additionally, birds exhibited improved gut-associated energy supply mechanisms, with enhanced carbohydrate metabolism and gut integrity. This indicates that *Bacillus* spp. helps to maintain a stable microbiota under pathogenic stress. Apart from gut microbiota modulating effects, *Bacillus* spp. are also studied for their direct antimicrobial effects. For instance, certain strains of *B. amyloliquefaciens*, *B. licheniformis or B. subtilis* produce bioactive compounds capable of inhibiting pathogens such as *Enterococcus cecorum*, further contributing to its protective role in gut health (Medina Fernández et al., 2019).

In swine, *B. subtilis* alone or in combination with *B. licheniformis* exhibited varied effects on microbiota composition. The effects of *Bacillus* on microbial diversity appear to be context-dependent, with some studies reporting decreased species richness (He et al., 2020a), while others find no significant changes (Kaewtapee et al., 2017; Luise et al., 2019; Wang et al., 2021; Jinno et al., 2022). In standard conditions, the supplementation with *B. subtilis* has been reported to increase beneficial microbes such as *Bifidobacteria*, *Ruminococcaceae*, and *Lactobacillaceae*, particularly in the jejunum and colon in weaning and growing pigs (Kaewtapee et al., 2017; Jinno et al., 2022). Interestingly, certain microbial shifts were associated with improved nutrient absorption and metabolic functions, highlighting the role of *Bacillus*-based probiotics in modulating gut homeostasis (Wang et al., 2021). Under challenging conditions, such as *E. coli* F4ac and F18 infection, *B. subtilis* strains such as DSM 25841 or DSM 32540 has been shown to reduce *Enterobacteriaceae* while promoting microbial stability (Luise et al., 2019; He et al., 2020b; He et al., 2020a).

In both poultry and swine, *Bacillus*-based probiotics spp. may exert cross-feeding effects and modulate hindgut fermentation processes, which can lead to increasing levels of short-chain fatty acids (SCFAs) while decreasing harmful metabolites such as ammonia and biogenic amines. Such a change in fermentation profile will support intestinal functions and reduce inflammation (Khan and Chousalkar, 2020). These findings suggest that *B. subtilis* supplementation can support gut health by selectively enhancing beneficial bacteria and reducing pathogenic populations in both poultry and swine.

### 3.3 Gut epithelium

A good body of intestinal morphological traits, including villus height, crypt depth and villus-to-crypt ratio are considered markers of gut health (Rysman et al., 2023). Better proliferation of enterocytes leads to longer villi and therefore to an increased intestinal surface area, which improves nutrient absorption, feed conversion ratio, and average daily gain in broilers. This is also confirmed by an enhanced better total tract digestibility of dry matter, organic matter, gross energy and crude protein (He et al., 2019), higher activities of digestive enzymes, including trypsin, amylase, lipase, and total protease (Gong et al., 2018). The use of *Bacillus* probiotics has been linked to a better-established intestinal morphology with longer villi and microvilli under standard production stress (Jacquier et al., 2019). Also in challenge conditions such as heat stress (Abdelqader et al., 2020) and subclinical necrotic enteritis challenge (Wang et al., 2020), dietary supplementation of *Bacillus*-based probiotics could partially restore challenge-related growth impairment of intestinal villi.

Apart from an active proliferation of gut epithelial enterocytes, it is crucial that these cells are tightly adhered to one another, to form a protective intestinal barrier that prevents the translocation of bacteria and toxins from the lumen to the bloodstream. Studies have shown that supplementing animals with *Bacillus*-based probiotics, either in combination (e.g., *B. subtilis*, *B. licheniformis* and *Saccharomyces cerevisiae*) or as a single strain such as *B. subtilis* DSM 29784 have resulted in an increased intestinal expression of tight junction-related genes, such as *claudin-1* (CLDN1), *occludin* (OCLN) and *Zonula occludens*-1 (ZO-1) (Ariyadi and Harimurti, 2015; He et al., 2019), which leads to a better intestinal barrier function under normal and inflammatory conditions (Rhayat et al., 2019).

The number of goblet cells is also to be considered as a marker for gut function, as their abundance is a good indicator of intestinal activity (Gustafsson and Johansson, 2022), mainly due to their role in producing mucin, which in turn coats the inner surface of the lumen and restricts the adherence of pathogens (Shroyer and Kocoshis, 2011; Gong et al., 2018; Abdelqader et al., 2020). Dietary supplementation of *Bacillus* probiotics (such as *B. coagulans*) increased goblet cell numbers (Zhen et al., 2018) and mucin production, which might be linked to a higher release of prostaglandin (Kunikata et al., 2002).

So, it is imperative to note that dietary *Bacillus*-based probiotics supplementation has a significant role in contributing to a better development and function of the gut epithelium, resulting in better nutrient absorption and better defense against pathogens (Fathi et al., 2018; Gong et al., 2018; Abdelqader et al., 2020; Ebeid et al., 2021).

### 3.4 Gut-associated immune system

One of the mechanisms of action of *Bacillus*-based probiotics is their influence on mucosa-associated lymphoid tissue (MALT), which includes the Harderian glands, bronchial-associated lymphoid tissue, nasopharyngeal lymphoid tissue, and gut-associated lymphoid tissue (GALT). Among these, GALT plays a crucial role in immune development, rapid pathogen response, and even endocrine and neural modulation.

GALT is a key component of the mucosal immune system, defending against pathogens entering via the gastrointestinal tract. Its primary structures include Peyer’s patches, cecal tonsils, mesenteric lymph nodes, and dispersed lymphocytes in the lamina propria and intestinal epithelium (Casteleyn et al., 2010; Mabbott et al., 2013). While swine show well-developed lymph nodes and continuous ileal Peyer’s patches, chickens lack true lymph nodes and rely more heavily on cecal tonsils and the bursa of Fabricius. These anatomical distinctions reflect species-specific adaptations in mucosal immune function and lymphoid tissue development (Casteleyn et al., 2010; Jørgensen et al., 2022).

*In vitro* and *ex vivo* studies indicate that *Bacillus* strains directly interact with GALT-associated immune cells. *B. subtilis* DSM 32315 and *B. velezensis* CECT 5940 improve T cell activities: assessed by increased counts of activated T lymphocytes (CD4^+^ CD25^+^, CD8^+^ CD25^+^ and CD28^+^ phenotypes) and enhance interleukin-10 (IL-10) production, a key anti-inflammatory cytokine (Larsberg et al., 2024). These bacteria also promote dendritic cell and macrophages antigen presentation functions, fostering an efficient immune response (Benson et al., 2012). Additionally, treating Caco-2 cells with *B. subtilis* DSM 29784 or their secreted metabolites, whether in spore or vegetative form, can reduce NF-κB and AP-1 proinflammatory signaling pathways and IL-8 production in a strain-dependent manner, with vegetative cells exhibiting enhanced immunomodulatory activity (Rhayat et al., 2019; Vieco-Saiz et al., 2024). Although spores show lower activity, their effects may be attributed to bioactive proteins and enzymes present in the spore crust (Mazzoli et al., 2019; Koopman et al., 2022).

In monogastric animals, *Bacillus* probiotics can be triggered by direct immunomodulatory effects and indirect reshaping of the gut microbiota (see above). Hence, their supplementation in broilers is associated with increased caecal abundance of butyrate-producing bacteria and higher density of intraepithelial lymphocyte (IEL) populations (Jacquier et al., 2019). These anti-inflammatory effects are especially evident under pathogen challenges, such as with *Salmonella* or *C. perfringens*, where *Bacillus* supplementation mitigated acute inflammatory responses (Keerqin et al., 2021; Kulkarni et al., 2022). It is noteworthy that these effects do not preclude immune activation. *Bacillus* spp. such as *B. subtilis* BG5 or BYS2 can simultaneously enhance IgA production, macrophage phagocytic activity, and nitric oxide synthesis (Guo et al., 2020; Jiang et al., 2021). Despite the lack of literature on *Bacillus* species effects on swine GALT structures, multiple *in vivo* studies suggest a modulatory role in mucosal immunity. Strains of *B. subtilis* and *B. licheniformis* such as PB6 have been shown to downregulate pro-inflammatory biomarkers such as TNF-*α*, IL-6, and TLR-4 in ileal tissues and intestinal epithelial cells (Aperce et al., 2010; Liu Y. et al., 2025), contributing to a balanced mucosal immunity. Thus, the reductions in inflammatory markers and bacterial translocation to mesenteric lymph nodes in pigs challenged with *E. coli* F18 support the notion that *Bacillus* supplementation may influence GALT-related immune responses indirectly through mucosal signaling and immune cell recruitment as (He et al., 2020a) demonstrated for *B. subtilis* DSM 32540 and *B. pumilus* DSM 32539.

In summary, *Bacillus*-based probiotics modulate GALT through both direct immune cell interactions and indirect effects via microbiota regulation. These actions enhance the hosts’ immune competence by improving the immune response, regulating the inflammation, and supporting the mucosal protection against gastrointestinal pathogens.

### 3.5 Extra-intestinal effects

The physiological and microbiological processes occurring in the gut can affect the health and functions of organs outside the intestinal tract. This is obvious for pathogenic bacteria that translocate from the intestine to other organs and cause diseases, such as enterococcal spondylitis (Wideman, 2016). However, over the last decades, it has become increasingly clear that, aside from these disease-related unidirectional effects, animal and human health depends on a more continuous and bidirectional communication setup between the digestive tract and several extra-intestinal organs. In the paragraphs below, we will briefly discuss the gut-liver, gut-lung, and gut-brain axes.

#### 3.5.1 Gut-liver axis

The gut and liver communicate via the circulatory system, biliary tract, and the portal vein, which directly transports gut microbiota-derived nutrients, bacterial components, and metabolites to the liver, profoundly impacting hepatic function and metabolism (Wahlström et al., 2016). For instance, SCFA produced by hindgut bacteria can induce intestinal gluconeogenesis and trigger sensors in the portal vein, thereby regulating hepatic metabolism, fat storage, and insulin sensitivity (De Vadder et al., 2014).

The gut-liver axis is often studied under challenging conditions like ethanol exposure and heat stress. Heat stress in poultry and swine can compromise the intestinal barrier, allowing bacterial components like LPS and DNA to enter the portal vein, potentially overwhelming liver immune defenses and causing systemic endotoxemia (Ringseis and Eder, 2022). While *Bacillus*-based probiotics have been described to improve gut and liver health and affect behavior in heat-stressed broilers (section SDG 12 below), studies in challenged swine often focus on the gut and oxidative status, without addressing liver function (Ringseis and Eder, 2022).

Additionally, the gut-liver axis is studied in the context of fat metabolism in poultry, as the liver is the primary site of *de novo* lipogenesis. In older laying hens, gut dysbiosis can aggravate ageing-related liver fat accumulation, reduction of egg quality, and risk of steatohepatitis (Hamid et al., 2019). Changes in the hen diet affecting gut bacteria have been suggested to regulate hepatic lipogenesis through metabolite-driven changes in gene expression (Chen C. et al., 2023). Probiotic interventions are therefore being explored to combat excessive liver fat deposition in poultry. For example, in ducks, fermented feed with *Bacillus*, *Lactobacillus*, and yeast altered the abundance of intestinal bacterial families like *Ruminococcaceae* and *Lachnospiraceae*, improving gut health and reducing hepatic fat storage (Gu et al., 2022).

#### 3.5.2 Gut-lung axis

The proper maturation of gut microbiota is of prime importance to develop and control the gut-associated immune system (Thompson-Chagoyán et al., 2005). In addition, it becomes increasingly clear that intestinal bacteria can also modulate the natural defense systems of organs outside the digestive system, such as the lungs (Saint-Martin et al., 2022).

The existence of a functional gut-lung axis in swine is supported by studies showing that respiratory viral infections can affect the gut microbiome (Zhao et al., 2021) and vice versa (Niederwerder, 2017). Probiotics can work via the gut-lung axis. For example dietary supplementation with *B. subtilis* 597 has been shown to reduce lung pathologies during influenza infection (Winther et al., 2024) as well as in a co-infection model involving Porcine Reproductive and Respiratory Syndrome Virus (PRRSV) and *Salmonella* where a mixture of 6 *B. subtilis* strains improved outcomes (Zuckermann et al., 2022).

Evidence for a functional gut-lung axis has been described in poultry as well. Saint-Martin et al. (2024) found evidence for the gut microbiota modulating pulmonary metabolite levels, immunity-related gene activity and antiviral mucosal responses against avian influenza. In a *Mycoplasma gallisepticum* challenge model, chickens receiving a dietary *B. subtilis* KC1 had less pathological lung lesions, and their gut microbial dysbiosis, disrupted indole levels and pro-inflammatory cytokine levels were restored (Chen X. et al., 2023).

#### 3.5.3 Gut-brain axis

The gut-brain axis links the gastrointestinal tract with the central nervous system. The communication between these two organs relies on neural, endocrine, and immunity-associated pathways, as well as bacterial components and metabolites related to the gut microbiota (Sampson and Mazmanian, 2015). Therefore, dietary interventions such as probiotic supplementation have the potential, at least in theory, to modulate the brain and stress-related and other behaviors.

In rodent models of Parkinsons’s disease and neuroinflammation, *Bacillus* probiotics (e.g., *B. velenzensis* ADS024 or *B. subtilis* PXN21) have been described to improve gut microbial dysbiosis, neuronal function and survival and to modulate receptor expression of white blood cells (Acton et al., 2024; Rout et al., 2024). In rats and mice subjected to chronic mild stress, *Bacillus* probiotic strains have been reported to improve villi morphology, change the gut microbial composition and metabolite profile, stress hormones in the blood and neurotransmitter levels in the brain. These effects have been observed with probiotic products containing a mixture of 4 strains of *B. clausii* (SIN, T, O/C and N/R); *B. licheniformis* or *B. coagulans* IS-2 (Rehman et al., 2022; Feng et al., 2023; Satti et al., 2023).

Gut-brain research in livestock animals is less advanced, but in recent years, studies are being published demonstrating that in poultry, similar inter-organ communication mechanisms are conserved. For example, heat-stressed broilers supplemented with a mixture of three *B. subtilis* strains showed reduced levels of pro-inflammatory molecules in the hippocampus (Fu et al., 2023), or different bacilli, such as *B. subtilis* natto or *B. licheniformis* or *B. cereus*, *were able* to reduce heat stress-related behavioral responses while improving active foraging (Wang et al., 2018).

In summary, *Bacillus* strains can secrete bioactive molecules that modulate the function of both intestinal and extra-intestinal tissues, which can explain their potentially supporting effect on animal health and performance. In the following section, we will discuss how this can contribute both directly and indirectly to sustainable development.

## 4 Sustainable development challenges tackled with *Bacillus*

To explain how *Bacillus*-based probiotics can support various aspects of livestock production, their possible applications are discussed in the context of four out of the 17 sustainable development goals (SDGs), established by the United Nations in 2015.

### 4.1 SDG 2—zero hunger

About 8.9% of the world population suffered from hunger in 2022 (Arora and Mishra, 2022). Moreover, the rising global population will drive a greater demand for animal protein, necessitating increased livestock production. Poultry and pork are key contributors to global meat consumption, accounting for 40 and 34%, respectively, in 2022. Over the past three decades, both sectors have experienced significant growth, with pork consumption increasing by 77% and poultry by 287%. As demand continues to rise, both industries will play a crucial role in ensuring a sustainable and sufficient food supply (FAOSTAT, 2022; OECD, 2022; Kim et al., 2024). Hence, solutions to optimize animal performance, allowing optimal growth and high feed efficiency, are necessary to ensure achieving Sustainable Development Goal “Zero hunger.”

In swine the beneficial effects of *Bacillus* have been investigated, for instance, by Wang et al. (2019), *B. subtilis* GCB-13-001 provided to weaned piglets resulted in significantly higher body weight at 7, 21 and 42 days after weaning. As average daily feed intake was not influenced by *B. subtilis* GCB-13-001 in this trial, the overall FCR was significantly improved. Hu and Kim (2022) reported significant increased body weight, average daily gain, and FCR after supplementing weaned piglet diets with *B. subtilis* C-3102 spores. Also, in challenged conditions with *E. coli*, *B. subtilis* was able to significantly improve body weight of weaned piglets 28 days after weaning. Overall, feed intake was not influenced, resulting in a significant improvement in feed conversion ratio (FCR) (He et al., 2020b). A meta-analysis revealed that also in growing-finishing pigs average daily feed intake was not influenced by *Bacillus* spp. supplementation. Average daily gain tended to be increased and the FCR was significantly improved with *Bacillus* spp. (Gonzalez-Ronquillo et al., 2022).

In broilers similar results have been obtained, for instance, Bai et al. (2017) reported significant increased final body weight and average daily weight gain over a 42-day trial period when *Bacillus subtilis* fmbJ was supplemented to the diet at three different dose levels. The two higher dose levels did not influence feed intake, whereas the lowest dose level significantly increased feed intake. Hence, FCR was only significantly improved with the two highest dose levels. Furthermore, significant improvements of meat quality parameters were found, which was in line with several other studies (Zhou et al., 2010), but also in contrast to other research where no beneficial effects on meat quality could be observed after *Bacillus* supplementation (Zhang et al., 2012). It should be noted that also contradictory results on performance have been found. Nevertheless, the meta-analysis conducted by Ogbuewu and Mbajiorgu (2022) revealed that feed intake was not significantly affected by *Bacillus* spp. supplementation; however, when the data was segregated by bird strain, feed intake was significantly improved for Cobb birds, but not for other breeds. FCR and average daily gain were both significantly improved compared to control which remained true when the data was segregated by breeds or *Bacillus* spp. This indicates that the results were not dependent on these specific parameters. The meta-analysis further substantiates the ability of *Bacillus* spp. to improve performance in poultry production.

### 4.2 SDG 3—health and well-being

High productivity demands of animals often result in increased risk of diseases and microbial infections. Antibiotics are generally used to cure animals, but with the high number of antibiotic-resistant bacteria alternative solutions must be found to guarantee both animal and human health. In weaned piglets, postweaning diarrhea, mainly caused by enterotoxigenic *Escherichia coli* (ETEC) is, for instance, commonly prevented and treated by antibiotics. However, supplementation of *B. licheniformis* PF9 showed to be able to significantly alleviate the severity of diarrhea induced by an ETEC challenge (Xu et al., 2024). Although antibiotics seem to cure diarrhea in piglets, the microbial composition remained close to the microbial profile of piglets with diarrhea. In contrast, supplying a mixture of three *Bacillus* strains cured diarrhea in piglets and restored the microbiota close to the composition of healthy animals, which is crucial to re-establish healthy conditions in piglets (Yue et al., 2020).

Another good example of a bacterial infection that is commonly treated with antibiotics is *Lawsonia intracellularis*, the causative agent of ileitis in pigs. *B. licheniformis* and *B. pumilus* were able to alleviate the macroscopic and microscopic lesions induced by a *L. intracellularis* challenge. Furthermore, these two strains were able to reduce intralesional *L. intracellularis* antigen levels and bacterial shedding. However, *B. amyloliquefaciens* was also evaluated in this trial, but it was not able to suppress the infection, indicating that there are strain-specific effects (Opriessnig et al., 2019).

A big health threat to the global pig industry is caused by African swine fever virus (ASFV), with mortality rates close to 100% and no safe commercial vaccine nor antiviral drug available. It has been shown that *B. subtilis* can inhibit several viruses like influenza virus (Starosila et al., 2017), porcine epidemic diarrhea virus (Yuan et al., 2018; Peng et al., 2019) and more recently, the potential effect against ASFV was investigated by Lv et al. (2023). Challenged piglets with ASFV showed reduced morbidity and mortality when they were fed with liquid biologics or powders derived from *B. subtilis*. Small-molecule metabolites from *B. subtilis* arctiin and genistein, are likely related to the antiviral activity, as they showed to compete for ATP binding to the ATP-binding domain of ASFV, suppressing the ASFV proliferation (Lv et al., 2023).

In poultry, *Bacillus*-based probiotics have also shown considerable efficacy in reducing the colonization of intestinal pathogens such as *Salmonella* spp. and *C. perfringens.* For instance, *B. coagulans* supplementation in chicks significantly alleviated intestinal damage and inflammation caused by *Salmonella Enteritidis*, enhancing mucosal barrier integrity through goblet cell differentiation and upregulation of immune-related factors such as IgA and avian beta-defensins (Xie et al., 2022). Similarly, *B. pumilus* TS1 was found to protect broilers from *Salmonella Enteritidis*-induced oxidative and inflammatory damage by modulating stress proteins (e.g., HSP70, HIF-1*α*) and reducing pro-inflammatory cytokines like IL-1β, IL-6, and TNF-α (Liu et al., 2023).

In the context of necrotic enteritis caused by *C. perfringens*, *Bacillus* probiotics, especially *B. subtilis*, demonstrated strong preventive effects. A meta-analysis by Ghimire et al. (2024) confirmed that dietary supplementation with *B. subtilis* significantly reduced lesion scores and improved FCR in NE-challenged broilers. Consistently, strain-specific studies have shown that, for instance, *B. subtilis* DSM 29874 supplementation enhances growth performance and gut health while reducing intestinal damage in NE-challenged broilers through modulation of tight junction proteins, immune markers, and beneficial microbiota (Rhayat et al., 2017; Keerqin et al., 2021). Kulkarni et al. (2022) supported these outcomes, highlighting that *Bacillus* species help to suppress *C. perfringens* growth, regulate inflammatory responses, and enhance tight junction protein expression, contributing to improved intestinal barrier function.

Beyond pathogen control, *Bacillus* probiotics also improved poultry welfare by mitigating clinical signs of enteric disease and related conditions. For example, supplementation with *B. velezensis* CE100 reduced *Salmonella* counts in the cecum and significantly lowered the incidence of pododermatitis, a key welfare concern in broiler production (Park and Sun, 2022). This improvement was associated with better litter quality, reduced moisture content, and increased beneficial lactic acid bacteria, suggesting indirect benefits of *Bacillus* on the rearing environment. Taken together, these studies support the role of *Bacillus* spp. as multifunctional probiotics that not only control pathogens but also contribute to overall poultry health and well-being.

### 4.3 SDG 12—responsible consumption and production

Attention to animal welfare in livestock production is increasing as consumers are becoming more conscious of the origins of their food, driving demand for ethically produced animal products. In addition, improved welfare practices, including stress reduction, lead to healthier animals, better quality products, and can foster more responsible and sustainable production practices.

Probiotics can support welfare by reducing diseases and infections, or maximizing tissue and organ health, thereby increasing animal resistance to production stress. In addition, evidence suggests that health-supporting additives can also modify animal behavior in ways that do not involve disease prevention, and that arguably arose as a secondary effect to their ability to change the gut environment (Johnson and Foster, 2018). Some examples of these effects are given below.

Certain *C. perfringens* strains induce enteritis in poultry, not only negatively affecting animal performance, but also triggering stress-related behaviors (Sadeghi et al., 2015). As discussed, *Bacillus-*based probiotics can improve the clinical outcome of necrotic enteritis (see SDG 3—health and well-being) but was also reported to protect against *C. perfringens*-related brain damage and aggressive behavior such as feather-pecking (Chen et al., 2024).

Pododermatitis, a chicken footpad infection, causes pain and limits movement, eating, and drinking. It originates from small foot wounds exposed to bacteria and ammonia in wet and sticky litter. Bad litter quality is therefore an important predisposing factor for pododermatitis. Contributing factors include temperature and humidity in the poultry house, high stocking density, diet composition, and intestinal health issues. *Bacillus* probiotics can improve digestive health, thereby reducing litter moisture and the prevalence of pododermatitis, thereby enhancing broilers’ welfare (Park and Sun, 2022).

During periods of heat stress, animals will be less active, as seen by a reduction in walking and foraging behavior. They will also spend more time drinking and trying to eliminate excessive body heat by spreading their wings, and panting. In heat-challenged broilers, supplementation of *Bacillus* probiotics has been reported to increase locomotor and foraging behavior (Wang et al., 2018). At the same time, it mitigated heat-induced rises in hepatic pro-inflammatory cytokines and caecal antibody concentrations.

Heat stress can also impair skeletal health (Jiang et al., 2021). Bone strength is important for welfare in production animals, especially broilers, as their rapid growth and significant muscle mass predispose heavy birds to leg disorders and fractures. *B. subtilis* supplementation, however, can significantly improve tibial strength in broilers and turkeys. This effect was demonstrated on 3 isolates: two *B. amyloliquefaciens* and one *B. subtilis* (Tellez et al., 2020) resulting in better mineral absorption (Mohammed et al., 2021) or control of heat-induced inflammation (Yan et al., 2020).

Pigs can exhibit behavioral problems as well, such as tail-biting due to production stress and the establishment of social hierarchies. Evidence for an effect of *Bacillus* probiotics on welfare is scarcer in swine as compared to poultry, but piglets challenged with *Salmonella* Typhimurium were reported to be less active, with a reduced explorative and eating behavior, which was attenuated when they were fed a diet containing a *B. licheniformis* probiotic (Barba-Vidal et al., 2017).

### 4.4 SDG 13—climate action

Several measures can be taken to mitigate the negative consequences of livestock production on sustainability and climate change, including maximizing the efficient uptake and use of feed materials, as well as facilitating the use of alternative and more sustainable raw materials (Vastolo et al., 2024). As feed utilization is linked to animal performance, which is discussed above (section SDG 2), we focus here on reducing fecal ammonia. Ammonia can not only lead to odor problems and eye and respiratory tract irritation, it also can be converted to nitrous oxide, which is an important contributor to global warming (Nordahl et al., 2023).

In swine, probiotics are evaluated for their ammonia-reducing capacity in two ways: either as a product to be applied directly to the manure, or as a probiotic in the feed. Both approaches make use of the diverse metabolic activity of *Bacillus* species to induce chemical modification of nitrogen-containing compounds. Ammonium-tolerant strains can be screened for their ammonia-reducing activity *in vitro* (Shen et al., 2023), or evaluated when incubated with manure, which can result in a substantial reduction of ammonia and other volatile organic compounds (Kuroda et al., 2015; Kuroda et al., 2017; Hwang et al., 2023). When applied as a dietary supplement in pig diets, different *Bacillus*-based probiotics can modulate the gut microbial composition, enhance nitrogen digestibility, increase fecal *Lactobacillus* counts and decrease methane and/or ammonia emission (Payling et al., 2017; Prenafeta-Boldú et al., 2017; Lan and Kim, 2019; Fang et al., 2024).

Although in poultry as well, *Bacillus*-based probiotics have been investigated as direct litter management tool (Zhao et al., 2024), research mainly focused on its probiotic application to reduce fecal ammonia. Broilers fed with *Bacillus* probiotics (e.g., *B. amyloliquefaciens* KB3, *B. subtilis* C3102, or a combination of *B. subtilis* RX7 and B2A) were reported to have an improved nitrogen digestibility, resulting in reduced emissions of ammonia and other noxious compounds, such as hydrogen sulfide (Ahmed et al., 2014; Jeong and Kim, 2014; Park and Kim, 2015; Biswas et al., 2023).

Interestingly, probiotics are also studied as a complementary strategy to other nutritional strategies aiming to reduce noxious gas emissions, such as lowering the dietary levels of metabolizable energy and crude protein in broilers (Upadhaya et al., 2019) or the use of alternative raw materials in feed. For example, the use of distillers dried grains with solubles (DGGS) had advantages in terms of sustainability, as it upcycles cheap byproducts of ethanol production. However, the use of DGGS can lead to increased methane and nitrogen-containing emissions (Jarret et al., 2011; Trabue and Kerr, 2014). In laying hens, probiotic *B. subtilis* mitigated the DGGS supplementation-induced increase of nitrogen and phosphorous excretion (Abd El-Hack et al., 2017).

These examples demonstrate that *Bacillus*-based probiotics can improve nitrogen retention and reduce ammonia emissions in swine and poultry, thereby helping in making animal production more sustainable and more climate-friendly.

## 5 Discussion and future research

### 5.1 Documentation mode of action

While the oral route remains the most common method for probiotic administration, alternative delivery strategies such as spray, litter, and *in ovo* methods are gaining attention. *In ovo* administration of *B. subtilis* has demonstrated benefits for immune function, metabolic efficiency, and bone development, while also reducing oxidative stress and improving liver function (Oladokun and Adewole, 2023). Beyond gut health, different species of *Bacillus*, such as *B. subtilis*, *B. licheniformis*, and *B. indicus* are being explored for respiratory disease prevention, with spray delivery showing promise in strengthening immune defenses against avian influenza (H9N2) (Rasaei et al., 2023).

Additionally, *Bacillus* spores function as vaccine adjuvants, enhancing immune responses through increased virus-specific IgG production and T-cell activation (Lee et al., 2020). Looking ahead, genetically engineered *Bacillus* strains could offer more targeted health benefits by enhancing antimicrobial activity, modulating immune responses, or serving as live vaccine vectors. Examples include *B. subtilis* expressing defensin to prevent *Salmonella* Infantis infection; engineered strains carrying antigens like the PCV2 capsid protein (*B. subtilis*-Cap, *B. subtilis* WB600/ZD), which has shown superior immunogenicity in piglets compared to traditional vaccines (Zhang et al., 2020; Li et al., 2025) or carrying antimicrobial peptides such as cNK-2 against *Eimeria* (Wickramasuriya et al., 2023).

These advances highlight the potential of *Bacillus*-based probiotics as a next-generation tool for disease prevention. However, to make stronger claims related to health promotion, a better understanding is needed of the potential probiotics have in commercial production systems, but also of their limitations. This will likely necessitate more comprehensive evaluations of the protective and restorative properties of probiotics under challenging yet commercially relevant conditions, moving beyond merely descriptive analyses of cellular and gut microbiota changes linked to dietary probiotic supplementation.

### 5.2 Germination and growth dynamics in the gut

*Bacillus* spores in feed must germinate in the animal’s gut to become active vegetative cells that produce beneficial metabolites. Germination is a complex process that depends on several signaling pathways (Setlow, 2014) and external factors, such as nutrient availability, temperature, pH level, water activity, and the *Bacillus* strain’s genetics (Zhang et al., 2025). *Bacillus* spores have been demonstrated to germinate and multiply in the small intestine of mice (Casula and Cutting, 2002) and humans (Colom et al., 2021).

In day-old chicks orally gavaged with *B. subtilis* SC2362 or PHL-NP122, spore germination was detectable throughout the gastrointestinal tract within five to 24 h (Cartman et al., 2008; Latorre et al., 2014). However, in both studies, both spores and vegetative cells in the digestive tract declined to background levels by 120 h. These findings confirm that *Bacillus* does not colonize the intestinal tract and suggest that continuous supplementation of spores during the production cycle is required for sustained probiotic benefits. In chicks that were continuously fed *Bacillus* spores via sterilized feed, approximately 10% of the supplemented spores were recovered from the digesta of different gut segments, suggesting a germination rate of around 90% (Latorre et al., 2014).

Similarly, in pigs, *Bacillus* spores were detected throughout the gastrointestinal system after two weeks of probiotic supplementation, with an estimated 70–90% of spore germination in the proximal part of the digestive tract (Leser et al., 2008). Upon withdrawal, fecal secretion of spores and vegetative cells decreased gradually to the background level within one week (Leser et al., 2008).

To investigate the germination characteristics of probiotics products, researchers often rely on *in vitro* experiments such as growth curve analyses or more sophisticated setups mimicking the gastrointestinal tract conditions (Déat et al., 2009; Dufourny et al., 2019; Keller et al., 2019). However, whether these models accurately predict the dynamics of probiotics *in vivo*, is far from clear. A validated *in vitro* model to describe *Bacillus* behavior in livestock would be invaluable to evaluate the potential of probiotics in livestock production, as slight variations in their genetics can have profound effects on vegetative cell growth and function (Lu et al., 2021). In addition, factors like feed composition, gut content viscosity, feed retention time and the intestinal levels of antimicrobial peptides and SCFA will likely have a strong impact on *Bacillus* spore germination, growth, and metabolite secretion, and need to be studied in more detail. There is a need to develop *in vitro* models that do not only more closely mimic the physiological conditions of specific gastrointestinal regions of chickens and swine, but that are also validated to correlate with *in vivo* results.

In addition, tools need to be developed to monitor the behavior of *Bacillus* strains *in vivo* in a variety of intestinal conditions. This will add understanding of their modes of action and will open the door to developing dietary and other interventions to maximize their efficacy in livestock production.

### 5.3 Need for biomarkers

Many biomarkers are commonly used to assess gut health, but they do not specifically capture the effects of *Bacillus*. For example, dysbiosis is assessed by measuring the microbiota diversity and/or the abundances of certain taxonomic groups related to pathological or beneficial process (Manichanh et al., 2006; Reese and Dunn, 2018; Hagerty et al., 2020). An increase in Proteobacteria abundancies, for instance, is often linked to gut inflammation, while a reduction in butyrate-producing bacteria is associated with impaired gut health (Antonissen et al., 2016). Probiotic supplementation can result in mitigation of these microbiota imbalances, but the extent to which such changes in gut health biomarkers reflect direct or indirect effects of the probiotic products, often remains unclear.

Biomarker readouts that could be more directly related to the probiotic effect include pathogen suppression, such as the reduction of *C. perfringens* or *Salmonella* in feces and organs, as well as modulation of immune and barrier function responses, like villus height, crypt depth and the villus/crypt ratio, MUC2 expression, enhanced tight junction integrity, and downregulation of pro-inflammatory cytokines (Ducatelle et al., 2018).

More indirect biomarkers can arise from probiotic-triggered cross-feeding interactions, including gut microbial fermentation products (SCFAs, branched-chain fatty acids, biogenic amines), microbiota shifts as quantified by qPCR, e.g., *Lactobacillus/Enterobacteriaceae* ratio, or broader microbial modulation assessed through 16S rRNA sequencing. For example, the high levels of *Faecalibacterium* is often considered a positive biomarker of gut health (Machiels et al., 2014; Lopez-Siles et al., 2017; Martín et al., 2023). Additionally, it is crucial to consider that probiotic effects are strain-specific. The ability of *Bacillus* spp. to produce different metabolites and bioactive compounds varies by strain, influencing how they interact with the host and the microbiota. These strain-specific effects might also be one of the reasons why results in probiotic research lack consistency.

Given the diversity of *Bacillus* strains investigated and the broad spectrum of microbial and cellular effects they can induce, it is unlikely that a single readout or biomarker could fully capture the overall impact or elucidate the mechanisms of action of *Bacillus*-based probiotics.

Understanding probiotic mechanisms becomes even more challenging when multiple strains are combined, as their interactions may be synergistic, or antagonistic. Therefore, future research should focus more on exploring the mechanistic interactions among different probiotic strains and how these relationships influence host responses. In summary, despite promising insights, there is still a lack of standardization in biomarkers directly linked to *Bacillus*-based effects. A multi-faceted approach, combining metabolite profiling, microbiota assessment, and functional digestive markers, could provide a more comprehensive, dynamic and reliable evaluation of strain-specific probiotic benefits. Microbiome-based biomarkers represent promising non-invasive tools for assessing probiotic effects, given the microbiome’s pivotal role in maintaining health and influencing disease through its modulation of inflammatory and immune responses (Guo et al., 2022).

### 5.4 Extra-intestinal effects

Despite mounting evidence of endogenous and probiotic intestinal bacteria affecting the function of extra-intestinal organs, studying the communication axes the gut participates presents certain challenges. An important restriction in many studies describing probiotic-induced changes in the gut and in other organs, is the difficulty to separate causation and correlation in various signaling pathways. While it is reasonable to assume that the primary effects of in-feed supplemented probiotic spores are initiated in the gut, secondary mechanisms dependent on communication axes between the gut and extra-intestinal organs are multifaceted. These higher-order biological responses instigated by probiotics are therefore prone to considerable variation, as they depend on the physiological status, activity, and interconnectivity of other organs. This complexity makes it difficult to isolate, study and predict specific effects of probiotics outside the digestive tract.

## 6 Conclusion

The use of *Bacillus*-based probiotics in livestock production presents a promising avenue for enhancing animal health, growth performance, and welfare. Probiotic *Bacillus* strains have been shown to modulate nutrient absorption, gut microbiota, and immune function, with beneficial effects inside and outside the digestive tract of poultry and swine.

However, the current body of evidence is limited by notable heterogeneity. Differences in *Bacillus* strains, environmental and management conditions, and methodological factors such as sequencing platforms and statistical power complicate comparisons across studies. Addressing these limitations through standardized designs and improved reporting, along with the development of more representative *in vitro* models and reliable biomarkers to evaluate probiotic effects in vivo, will greatly strengthen future research.

Despite these challenges, the potential of incorporating *Bacillus* probiotics into livestock diets is becoming increasingly clear, making them a valuable tool for modern and more sustainable animal production practices.
